# A new polyvinyl alcohol hydrogel vascular model (KEZLEX) for microvascular anastomosis training

**DOI:** 10.4103/2152-7806.72626

**Published:** 2010-11-23

**Authors:** Tatsushi Mutoh, Tatsuya Ishikawa, Hidenori Ono, Nobuyuki Yasui

**Affiliations:** Department of Surgical Neurology, Research Institute for Brain and Blood Vessels-Akita, 6-10 Senshu-Kubota-machi, Akita 010-0874, Japan; 1 Ono and Co., Ltd., 2-12-5 Ginza, Chuo-ku, Tokyo 104-0061, Japan

**Keywords:** Microvascular anastomosis, neurosurgical training, polyvinyl alcohol hydrogel, vascular model

## Abstract

**Background:**

Microvascular anastomosis is a challenging neurosurgical technique that requires extensive training for one to master it. We developed a new vascular model (KEZLEX, Ono and Co., Ltd., Tokyo, Japan) as a non-animal, realistic tool for practicing microvascular anastomosis under realistic circumstances.

**Methods:**

The model was manufactured from polyvinyl alcohol hydrogel to provide 1.0–3.0 mm diameter (available for 0.5-mm pitch), 6–8 cm long tubes that have qualitatively similar surface characteristics, visibility, and stiffness to human donor and recipient arteries for various bypass surgeries based on three-dimensional computed tomography/magnetic resonance imaging scanning data reconstruction using visible human data set and vessel casts.

**Results:**

Trainees can acquire basic microsuturing techniques for end-to-end, end-to-side, and side-to-side anastomoses with handling similar to that for real arteries. To practice standard deep bypass techniques under realistic circumstances, the substitute vessel can be fixed to specific locations of a commercially available brain model with pins.

**Conclusion:**

Our vascular prosthesis model is simple and easy to set up for repeated practice, and will contribute to facilitate “off-the-job” training by trainees.

## INTRODUCTION

Microvascular anastomosis remains a challenging neurosurgical technique which requires extensive training for one to master it. Since clinical opportunities to experience such procedures on actual patients are limited, neurosurgeons are expected to develop and maintain their microsurgical skills with regular “off-the-job” practice alone. Typical laboratory microvascular training has been performed using artificial materials (such as gauze and silicone tube)[8,14] and *in vivo* (such as anesthetized rats)[7,10] and *in vitro* (such as swine and chicken-wing arteries)[6,18] animal models. Although these training modalities can certainly help trainees learn basic skills in microvascular anastomosis, each still has room for improvement in terms of simplicity, accessibility, repeatability, and life-likeness.

Polyvinyl alcohol (PVA) hydrogel has realized a biomodeling with mechanical properties similar to real cerebral vasculatures.[17] We introduce here a newly developed vascular model (KEZLEX #B61, Ono and Co., Ltd., Tokyo, Japan)[11] based on this material as a non-animal, user-friendly apparatus suitable for practicing microvascular anastomosis under realistic circumstances.

## MATERIALS AND METHODS

### Description of the model

**Figure 1:**
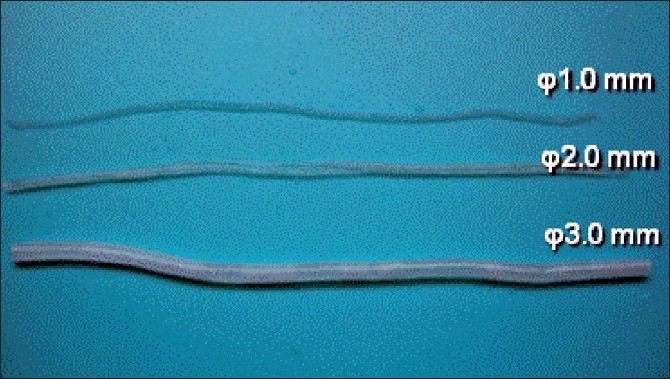
Photographs showing the new PVA hydrogel model for microvascular anastomosis training

**Figure 2:**
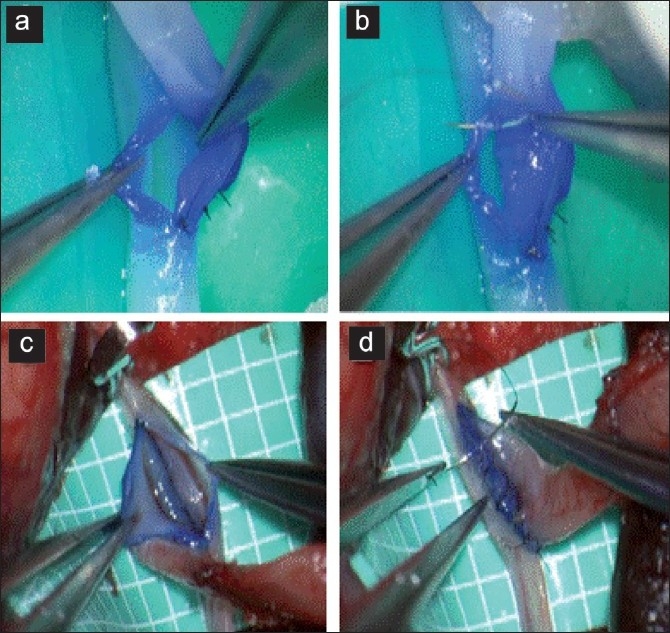
(a) The segment for arteriotomy can be colored with pyoctaninum blue dye for clear visualization of the plane of dissection/suturing. (b) End-to-side anastomosis with interrupted suturing. Note that the visibility of the walls, staining properties, and suturing feels of the model (a, b) closely resemble real human donor and recipient arteries (c, d)

The vascular substitute [Figure 1] was processed and extracted from PVA hydrogel by laser sintering technique to provide 1.0–3.0mm diameter (available for 0.5-mm pitch), 6–8cm long tubes that are qualitatively similar to human donor and recipient arteries for various bypass surgeries based on three-dimensional computed tomography/magnetic resonance imaging scanning data reconstruction using visible human data set and vessel casts. Both our pilot experiments and simulations of variable vascular pathologies by other groups[12,17] have shown that the PVA hydrogel vessel models exhibited similar mechanical properties (surface friction and elasticity) and visibilities (transparence and compatibility with staining to blue dye) to real arteries [Figure 2] that may be accepted as a good biomaterial for practicing microsurgical anastomosis. The prosthetic materials need to be kept moist by intermittent spraying with water to prevent structures from drying out during practice. The models are readymade and repeatedly usable for practicing. They are reusable for suturing practice, if kept moist in unsterilized tap water in room air, when not in use. Although any personal orders of modifications can be requested, manufacture price of this model (KEZLEX #B61) including a set of three tubes [Figure 1] is ¥9900 (corresponding to US$117, based on current Yen–Dollar exchange rate of about ¥85 per dollar). The advantages and disadvantages of currently available vascular models for practicing microvascular anastomosis *in vitro* are summarized in Table 1.

**Table 1:** Summary of the advantages and disadvantages of available vascular models for practicing microvascular anastomosis *in vitro*

	Advantages	Disadvantages
Human cadaveric head/brain	Anatomically the most realistic and the closest model to live surgery	Scarce opportunities for working with this model on individual basis: Educational programs like cadaver dissection course are required
	Brain infusion models have been described[1,19]	
Swine artery	Thicker but almost identical sizes to human vessels The feel of dissecting/suturing is like that for human vessels	Specific distribution route may need to be arranged, which may not be suitable for individual use Not readymade for practicing: Removal of the surrounding fat and connective tissue around the vessels are required before starting microvascular anastomosis
	Inexpensive if it can be purchased from a slaughterhouse as the neck vascular package Reusable by preserving it in glycerol[2,5]	Desirable sizes and lengths for each specific situation may not be harvested
Chicken-wing artery	Size and feel closely resemble those of human vessels Inexpensive and easily obtainable at the grocery store or from a farm[6,7]	Not readymade for practicing: Anatomical neurovascular dissection is required before starting microvascular anastomosis
	Infused model has been described[18]	
Silicone tube (Microvascular Practice card)[14]	Easy to obtain, set up, and repeatable Can be practiced at any time, in any place Less expensive Variable sizes and lengths can be chosen as needed	Different feel from real vessels (hard and inflexible without moisture)
PVA hydrogel tube (KEZLEX)	Qualitatively similar surface friction, transparency, and elasticity to real human vessels User-friendly (as described for the silicone tube)	Easily dries (needs to be kept moist)Short length (6–8 cm) More expensive than silicone tubes

For selecting an appropriate size for anastomosis training, a thinner tube of 1.0 mm in diameter may be used as a substitute for recipient cortical arteries (M4), intermediate size tubes of 1.5 mm in diameter for recipient cerebral arteries such as the insular segment (M2–M3) of the middle cerebral artery (MCA) and callosal segment (A2–A3) of the anterior cerebral artery (ACA), and thicker tubes of 2.0–3.0 mm in diameter to simulate donor arteries such as the superficial temporal artery (STA) and radial artery (RA).

## RESULTS

Trainees can acquire basic microsuturing techniques (i.e., gentle and precise preparation of the donor and recipient vessels and correct suture placement without penetrating the walls of other vessels) for end-to-end, end-to-side, and side-to-side anastomoses, with handling and visibility similar to that of real arteries. The segment for arteriotomy can be colored with methylrosaniline (pyoctaninum blue) dye for clear visualization [Figure 2]. The irregularity of the cut end at the arteriotomy site, as observed in real arteries, may be useful for practicing vascular trimming skill. Results of anastomoses are evaluated by direct observation of the inner wall cut under the microscope to search for kinking or strangulation.

**Figure 3:**
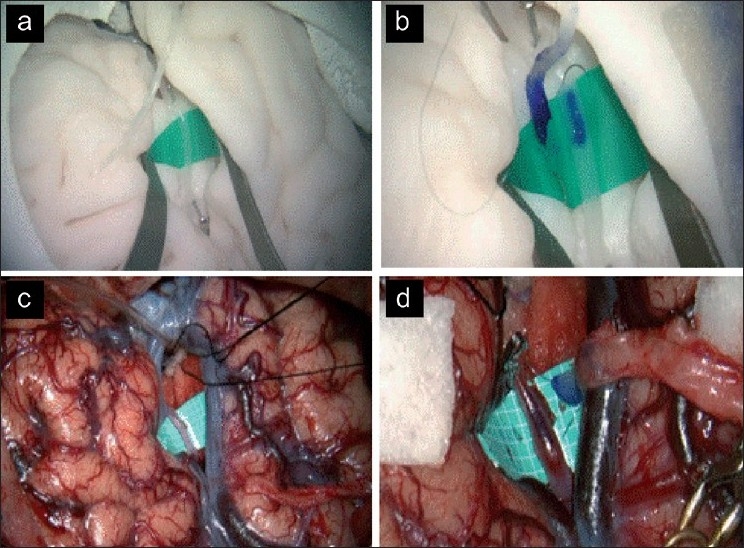
A set-up for practicing standard deep bypass (STA to M2). (a) To simulate a deep operative field, vascular models were placed onto the insular cortex of the artificial brain model with pins, a silicone sheet was inserted under the vessel, and the artificial brain was retracted with a brain spatula. (b) For preparation of end-to-side anastomosis, the prostheses were colored with pyoctaninum blue dye for clear visualization of the vessel walls as in actual surgery. Note that the views of the brain and vascular model (a, b) closely resemble real operative fields for microsurgical anastomosis (c, d)

Deep anastomosis has different levels of difficulty depending on depth. To practice standard deep bypass techniques to M2, the superior cerebellar artery, posterior cerebral artery, posterior inferior cerebellar artery, or callosal segment of the ACA as a recipient in realistic conditions, the substitute vessel can be fixed to specific locations of a commercially available brain model (KEZLEX #A36, Ono and Co., Ltd.)[9] with pins [Figure 3a and b]. Using this apparatus, practice can be performed in an operative field with not only realistic depth [Figure 3b and d] but also realistic width [Figure 3a and c] for manipulation of operative instruments.[4]

## DISCUSSION

Training in surgical skills is of great importance in mastering the techniques of microvascular anastomosis, which are indispensable in daily routine practice. Two major issues have been discussed in microsurgical training. The first involves the development of appropriate vascular substitutes. Gauze fibers,[8] silicone tubes,[14] synthetic vessels created from polyvinyl chloride[20] or polyurethane,[15] cadaveric materials,[1,19,21] and living and extracted animal vessels[6,10] have been reported as useful materials for training in anastomosis. Artificially pulsatile or perfused arteries in models have recently been reported to create relatively realistic situations.[1,3,13,18] However, these are neither convenient nor practical for individual use in daily practice, and a simple and accessible model that can be set up rapidly under safe and hygienic conditions and with no involvement of ethical questions is required. Our vascular prosthetic tube appears to have all of these characteristics, and trainees will be able to practice more easily and frequently under realistic suturing/handling conditions than with other non-biological vascular materials. In fact, recent data suggest that the PVA hydrogel vascular model has more similar pulsatility of vascular lumen to a real cerebral vasculature when compared to silicone,[17] although the compliance calculated from the diameter–pressure curve still needs room for improving.[12] In terms of the ability of structural modification in various sizes and shapes with KEZLEX technology[11] and of stiffness/elasticity of PVA hydrogel closer to the human soft tissue than silicone tube,[17] our model may have some advantages over existing “relatively harder” synthetic materials made from polyvinyl chloride[20] and polyurethane,[15,16] although direct comparison has not yet been performed.

The cost of the vascular prosthesis is another important factor for ideal training models. Unfortunately, current model is 1.5 times more expensive than commercially available microvascular practice model with silicone tubes fixed in a pocket-sized card (Microvascular Practice Card, Muranaka Medical Instruments Co. Ltd., Osaka, Japan).[14] Indeed, more efforts in cost reduction are still required but nonetheless, we believe that a main difference in texture and feel during manipulation with this model can provide a better idea for microsuturing real human vessels than classical silicone models.

The second issue is how situations of technical difficulty similar to actual vascular anastomosis can be reproduced. Deep microvascular anastomosis is a far more difficult technique than shallow STA–cortical MCA anastomosis, and few practical tools have been available for training in it. With installation of our vascular prostheses in brain models, we have demonstrated[9] variable depths of microvascular anastomosis (e.g., STA–M2, RA–M2, and A3–A3) can be practiced under realistic conditions. Using these models, demonstration of surgical techniques by an expert neurosurgeon is also possible even if no real patient is present.

Taken together, these considerations indicate that our new vascular substitute model will facilitate the development and maintenance of microsurgical skills in both resident neurosurgeons and experts who wish to master the various levels of microanastomosis techniques.
